# Supplementary material for: Patterns of Intron Gain and Loss in Fungi
Source: PLoS Biol. 2004 Nov 30;2(12):e422. doi: 10.1371/journal.pbio.0020422 (PMC532390; doi:10.1371/journal.pbio.0020422)
Supplement: Table S1 — Also available at http://genes.mit.edu/NielsenEtAl/. (4.3 MB ZIP). [file pbio.0020422.st001.zip › NielsenEtAl/html/856.html]

AN0687.1.NCU06727.1.MG06197.1.FG10249.1


```
 CLUSTAL W (1.82) Multiple Sequence Alignments - Introns Inserted


Sequence 1: MG06197.1	273 aa
Sequence 2: FG10249.1	294 aa
Sequence 3: NCU06727.1	291 aa
Sequence 4: AN0687.1	292 aa
Alignment Length: 295 aa
Number Identitical Residues: 195 aa
Alignment Score (without introns) 9014


MG06197.1 	-------------~--------MWPG1QAMTLKVEKVLHHERSKYQDVLIFQSTDYGKVL
NCU06727.1	MS--EIAHPTIQD1GWFREISNMWPG~QAMTLKVEKVLHHEKSLYQDVLIFKSTDHGNVL
FG10249.1 	MSNEEITHPTIVD1GWFREISDMWPG1HAMTLRVEKVLVHEKSKYQDVLIFKSTDFGNVL
AN0687.1  	MS--EITHPTIKD1GWFSEQSEMWPG~QAMNLRVNQILHHEKSKYQDVLVFESSDYGTVL
          	 :  . : .:  . .   . :.**** :**.*:*:::* **:* *****:*:*:*.*.**

MG06197.1 	VLDNVIQCTERDEFS~YQEMITHLAMNSHPNPKKVLVIGGGDGGVLREVVKHDCVEEAIL
NCU06727.1	VLDNVIQCTERDEFS~YQEMITHLAMNSHPNPKKVLVIGGGDGGVLREVVKHDCVEEAIL
FG10249.1 	VLDNVIQCTERDEFS~YQEMIAHLALNSHPNPKKVLVIGGGDGGVLREIVKHDCVEEATL
AN0687.1  	VLDNVIQCTERDEFS2YQEMITHLAMNSHPNPKKVLVIGGGDGGVLREVVKHETVEEAIL
          	*************** *****:***:**********************:***: **** *

MG06197.1 	CDIDE~AVIRLSKEYLPNMSAGFNHPKVKVHVGDGFKFLADYQNTFDVIITDSSDPEGPA
NCU06727.1	CDIDE0AVIRLSKQFLPHMSAGFEHPKVKVHVGDGFKFLEDFKNTFDVIITDSSDPEGPA
FG10249.1 	CDIDE~AVIRLSKEHLPSMACGFDHPKSKTHVGDGFKFLNDYKNEFDVIITDSSDPDGPA
AN0687.1  	CDIDE0AVIRVSKKYLPGMSIGFQHPNVKVHVGDGFEFLKQRQNEFDVIITDSSDPEGPA
          	***** ****:**:.** *: **:**: *.******:** : :* ***********:***

MG06197.1 	ESLFQKPYFQLLFNALTEGGVITTQ-A1ENQWLHLPLITKLRKDCKEIFPVAEYAYTTIP
NCU06727.1	ESLFQKPYFQLLHDALREGGVITTQ-A1ENQWLHLPLITKLKKDCKEVFPVAEYAYTTIP
FG10249.1 	EALFQKSYFQLLHDALREGGVITTQGS1ESPWLHLPLIARLKKDCGAIFPVAEYAYTTIP
AN0687.1  	ESLFQKPYFELLRDALRDGGVITTQ-A1ENQWLHLPLIADLKKACNEVFPVAEYAYTTIP
          	*:****.**:** :** :******* : *. *******: *:* *  :************

MG06197.1 	TYPSGQIGFMVCSKDPKANLKEPLRSWTQEEELEKCRYYSSDIHKASFVLPNFARKALQD
NCU06727.1	TYPSGQIGFMVCSKDPNANVKVPLRSWSQEEEEKLCRYYNSEIHKASFILPTFAKKALE-
FG10249.1 	TYPSGQIGFMVCSKDPKADVKNPIRSWTKEEEDEKLRYYSSEIHKASFVLPKFAAKALE-
AN0687.1  	TYPSGQIGFMVCCKDANRNVKEPVRTWSREEEERLCRYYNQDIHRASFVLPNFARKALGN
          	************.**.: ::* *:*:*::*** .  ***..:**:***:**.** *** .

MG06197.1 	
FG10249.1 	
NCU06727.1	
AN0687.1  	
          	
```
